# A role for the carbon source of the cell and protein kinase A in regulating the *S. pombe* septation initiation network

**DOI:** 10.1242/jcs.261488

**Published:** 2024-01-10

**Authors:** Özge Uysal Özdemir, Andrea Krapp, Bastien Mangeat, Marc Spaltenstein, Viesturs Simanis

**Affiliations:** ^1^ EPFL SV ISREC UPSIM, SV2.1830, Station 19, CH - 1015 Lausanne, Switzerland; ^2^ EPFL SV PTECH PTEG, SV 1535 (Bâtiment SV), Station 19, CH-1015 Lausanne, Switzerland

**Keywords:** Cell cycle, Septation initiation network, Cytokinesis, Respiration versus fermentation

## Abstract

The septation initiation network (SIN) is a conserved signal transduction network, which is important for cytokinesis in *Schizosaccharomyces pombe*. The SIN component Etd1p is required for association of some SIN proteins with the spindle pole body (SPB) during anaphase and for contractile ring formation. We show that tethering of Cdc7p or Sid1p to the SIN scaffold Cdc11p at the SPB, rescues *etd1-Δ*. Analysis of a suppressor of the mutant *etd1-M9* revealed that SIN signalling is influenced by the carbon source of the cell. Growth on a non-fermentable carbon source glycerol reduces the requirement for SIN signalling but does not bypass it. The decreased need for SIN signalling is mediated largely by reduction of protein kinase A activity, and it is phenocopied by deletion of *pka1* on glucose medium. We conclude that protein kinase A is an important regulator of the SIN, and that SIN signalling is regulated by the carbon source of the cell.

## Teaser

**Summary:** Signalling by the *S. pombe* septation initiation network, which regulates cytokinesis, responds to the change from aerobic fermentation to growth by respiration through reduction of protein kinase A activity.

## INTRODUCTION

*Schizosaccharomyces pombe* is a useful model for study of essential processes in eukaryotic cells (Fantes and Hoffman, 2016). Cells grow by tip-elongation and divide after forming a medial septum. A contractile actin ring (CAR) assembles at the division site during mitosis (Wu et al., 2003) and guides synthesis of the division septum (reviewed by Pollard and Wu, 2010). Coordination between mitosis and cytokinesis is assured by the septation initiation network (SIN) (Simanis, 2015). The SIN is essential for the formation (Hachet and Simanis, 2008; Huang et al., 2008) and maintenance of the CAR (Alcaide-Gavilan et al., 2014). If SIN signalling fails, growth and the nuclear cycle continue, but cytokinesis fails, producing multinucleated cells.

The SIN comprises three protein kinases, Cdc7p (Fankhauser and Simanis, 1994), Sid1p (Guertin et al., 2000) and Sid2p (Sparks et al., 1999), and their respective regulators Spg1p (Schmidt et al., 1997), Cdc14p (Fankhauser and Simanis, 1993; Guertin et al., 2000) and Mob1p (Hou et al., 2000; Salimova et al., 2000). Signalling is promoted by the GTPase Spg1p, which is controlled by its GTPase-activating protein (GAP) Cdc16p and the scaffold protein Byr4p (Furge et al., 1998; Minet et al., 1979; Song et al., 1996). The kinase Plo1p is important for septation and functions upstream of the SIN (Ohkura et al., 1995; Tanaka et al., 2001). These proteins associate with the spindle pole bodies (SPBs) via a scaffold comprising Cdc11p, Sid4p and Ppc89p (Chang and Gould, 2000; Krapp et al., 2001; Rosenberg et al., 2006; Tomlin et al., 2002).

Two states of the SIN are distinguishable during mitosis. In early mitosis, Cdc7p and Sid1p associate transiently with one or both SPBs (the ‘early’ SIN), whereas later, they associate only with the new SPB (the ‘late’ SIN) (Grallert et al., 2004; Guertin et al., 2000; Sohrmann et al., 1998; Wachowicz et al., 2015). The transition from the early SIN to the late SIN requires the anaphase promoting complex/cyclosome, with the Cdc20-family targeting subunit Slp1p (APC/C^Slp1^), Spg1p (Wachowicz et al., 2015) and Etd1p, which maintains Cdc7p at the SPB in anaphase (Daga et al., 2005; Garcia-Cortes and McCollum, 2009). Asymmetric distribution of SIN proteins requires silencing of Spg1p signalling on the old SPB by its GAP (Cerutti and Simanis, 1999; Li et al., 2000), and contributes to the fidelity of cytokinesis (Singh et al., 2011). Failure to remove Cdc7p from the SPBs at the end of mitosis (Fankhauser et al., 1993; Fankhauser and Simanis, 1994; Minet et al., 1979; Song et al., 1996) or tethering of Cdc7p to the SPB (Chen et al., 2017), deregulates septum formation, producing multiple septa, and promoting septation from anywhere in the cell cycle (Schmidt et al., 1997). Sid2p and Mob1p associate with both SPBs throughout mitosis and with the CAR during septation (Hou et al., 2000; Salimova et al., 2000).

In medium containing abundant glucose, *S. pombe* grows by aerobic fermentation and represses respiration. In low glucose media or on non-fermentable carbon sources, such as glycerol, cells switch to respiration (known as the Crabtree effect; Pfeiffer and Morley, 2014). Glucose is detected by the G-protein-coupled receptor Git3p, which activates Gpa2p (Isshiki et al., 1992). This stimulates adenylate cyclase (Cyr1p) to generate cAMP (Ivey and Hoffman, 2005; Welton and Hoffman, 2000), which promotes dissociation of protein kinase A (Pka1p) from its regulatory subunit Cgs1p (Byrne and Hoffman, 1993; Matsuo et al., 2008; Thevelein, 1984). *pka1* is not essential (Maeda et al., 1994) and strains with reduced Pka1p activity mimic glucose-starved cells (Hoffman, 2005a,b; Hoffman and Winston, 1991).

The cAMP/PKA pathway contributes to the regulation of many processes in fission yeast (Kelkar and Martin, 2015; Yamada et al., 1997; Yamashita et al., 1996). Overexpression of *pka1* arrests cells in G2 (Tallada et al., 2002), whereas cells lacking *cyr1* or *pka1* are advanced into mitosis (Kishimoto and Yamashita, 2000a,b; Navarro and Nurse, 2012). Reduction of Pka1p activity also promotes phosphorylation by Pak1p and Pak2p (Kunitomo et al., 2000; Prieto-Ruiz et al., 2023) of the type II myosin regulatory light chain Rlc1p (Le Goff et al., 2000; Naqvi et al., 2000), which becomes essential when cells are grown in glycerol (Malecki and Bähler, 2016).

In this study, we screened for suppressors of the loss-of-function *etd1* mutant *etd1-M9*. One of these mapped to *pdc101*, an isoform of pyruvate decarboxylase. Loss of Pdc101p rescues *etd1-M9* and *etd1-Δ.* Furthermore, growth on glycerol bypasses both the requirement for Pdc101p at high temperatures, and Etd1p for cytokinesis. This is mediated partly via Pka1p, implicating Pka1p as a regulator of SIN signalling.

## RESULTS AND DISCUSSION

The heat-sensitive mutant *etd1-M9* is viable at 19°C, whereas at 29°C and 32°C the cells become elongated and multinucleated (Fig. S1). *Etd1* is not essential at 36°C (Daga et al., 2005). *Etd1-M9* has multiple mutations (S301T, L305I, M383S and T384P) in its C-terminal domain, which is essential for its function (Alcaide-Gavilan et al., 2014).

**Fig. 1.**
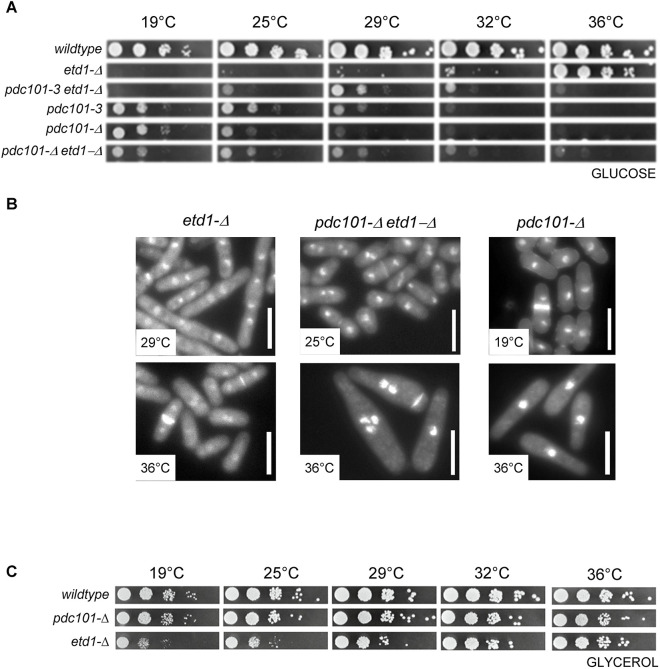
**The effect of carbon source upon colony formation by *etd1-Δ* and *pdc101-Δ* cells.** (A) The indicated strains were grown to exponential phase and dilutions were spotted on complete medium with glucose as the main carbon source at different temperatures. Note that the wild-type and *etd1-Δ* dilution series are duplicated in Fig. S2B. The intervening series shown in that figure are deleted in this one. The data shown in A and in Fig. S2B are derived from images taken of a single plate for each temperature. (B) Cells of the indicated genotypes were grown in GLU medium at 36°C (*etd1-Δ*), 19°C (*pdc101-Δ*) or 25°C (*etd1-Δ pdc101-Δ*), and shifted to the indicated temperatures (29°C, 36°C and 36°C, respectively, for the equivalent of two cell cycles). Cells were harvested, fixed and stained with DAPI and Calcofluor. Scale bars: 10 µm. (C) The indicated strains were grown to exponential phase and dilutions were spotted on complete medium with Glycerol as the main carbon source at different temperatures. Images in B and C are representative of three repeats.

We isolated spontaneous suppressors of *etd1-M9* that allowed division at 29°C. Sequencing of one of these mutants revealed a six base-pair insertion in SPAC1F8.07c*/pdc101* (Fig. S2A). A wild-type copy of *pdc101* rescued the heat-sensitivity of this mutant (data not shown) and linkage was observed with *isp3::KanMX6*, which is ∼4.5 kb from *pdc101* (Lock et al., 2019), producing 43 parental ditype (PD), 0 non-parental ditype (NPD) and 0 tetratype (TT) tetrads. We named the mutant *pdc101-3*. Given that *pdc101-3* is recessive (data not shown), it seems likely that it reduces Pdc101p activity. The double mutant *pdc101-3 etd1-M9* formed colonies better than *etd1-M9* at 25°C and 29°C, and *pdc101-3* was epistatic to *etd1-M9* at 36°C (Fig. S2B). *Pdc101-3* also rescued *etd1-Δ* at 29°C and was epistatic to *etd1-Δ* at 36°C (Fig. 1A).

At 36°C, *pdc101-3* cells arrested in the first cycle with a single nucleus (Fig. S2C,D). The cells displayed a morphology defect at 36°C; one end was wider than the other, and there was often curved growth at the thin end (Fig. S2D). Of the four pyruvate decarboxylase (*pdc*) genes, only *pdc101* is essential at 30°C (Hayles et al., 2013; Kim et al., 2014). Dissection of tetrads from *pdc101::G418^R^/pdc101^+^* revealed that *pdc101-Δ* grew at 19°C on YE medium plus 3% (w/v) glucose (hereafter GLU). As expected, *pdc101-Δ* was unable to form colonies on GLU at ≥29°C (Fig. 1A). Its phenotype at 36°C was similar to the *pdc101-3* mutant, although qualitatively, the cells curved less at the narrow end (Fig. 1B). The double mutant *pdc101-Δ etd1-Δ* was viable at 19°C and 25°C on GLU, and *pdc101-Δ* was epistatic to *etd1-Δ* at 36°C (Fig. 1A,B). *Pdc101-3 etd1-Δ* cells die at 19°C, presumably because Pdc101-3p retains some function at 19°C (Fig. 1A). We conclude that loss of Pdc101p activity bypasses the requirement for Etd1p on GLU.

*Pdc101* is the principal pyruvate decarboxylase expressed in exponential growth on GLU. Its expression decreases in stationary phase, under stress conditions (Kim et al., 2014; Wilhelm et al., 2008), and during the shift from glucose to glycerol (Malecki et al., 2016). Therefore, we tested whether growth on glycerol medium [YE medium plus 3% (v/v) glycerol and 0.1% (w/v) glucose; hereafter GLY] would rescue *etd1-Δ* and *pdc101-Δ*; both mutants grew on GLY at all temperatures (Fig. 1C).

**Fig. 2.**
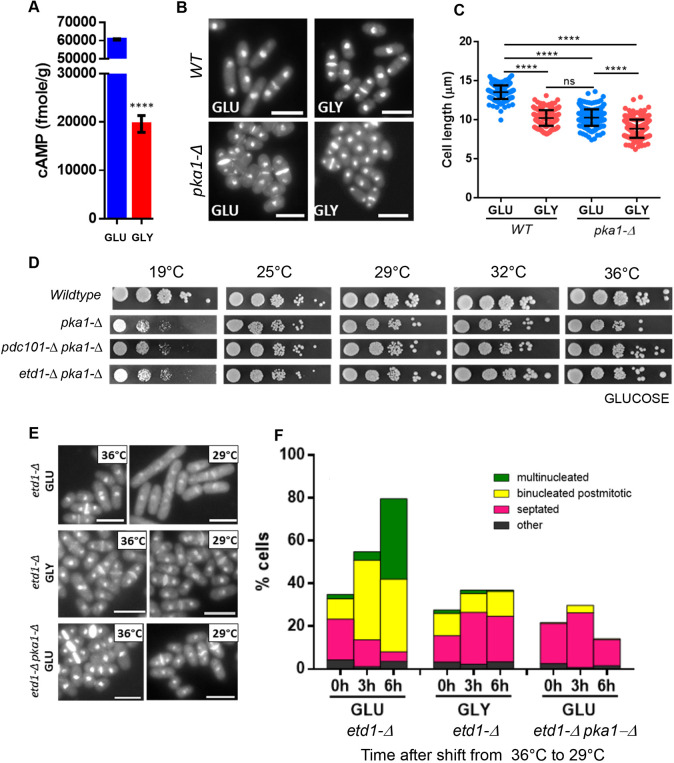
**Deletion of *pka1* rescues *etd1-Δ* and *pdc101-Δ* cells.** (A) Determination of cAMP concentration in cells grown in GLU or GLY. The mean of three independent experiments was used for plotting. Error bar are mean±s.d. (*n*=3). ****P<0.0001 compared with values of the cells grown in GLU medium (two-tailed unpaired Student's *t*-test). (B) Wild-type (WT) and *pka1-Δ* cells were grown in the indicated medium to exponential phase, harvested, fixed and stained with DAPI and Calcofluor. Scale bars: 10 µm. (C) Wild-type and *pka1-Δ* cells were grown in the indicated medium to exponential phase and the length of the septated cells was measured. The measurements of three independent experiments with >100 cells for each were pooled and plotted. Error bars with mean±s.d. ****P<0.0001; ns, not significant for the comparison of the means of each group (one-way ANOVA with Tukey post test). (D) The indicated strains were grown to exponential phase and dilutions were spotted on GLU medium at different temperatures. Images are representative of three repeats. (E) The indicated strains were grown to exponential phase in the indicated medium at 36°C, then shifted to 29°C. Cells were fixed and stained with DAPI and Calcofluor. Scale bars: 10 µm. (F) The indicated strains were grown to exponential phase at 36°C and then shifted to 29°C. The phenotypes were quantified after fixation and staining with DAPI and Calcofluor. The *x*-axis shows the time after temperature shift and the growth medium. Results are the mean for three repeats with 100 cells analysed per repeat.

As expected, the concentration of cAMP was reduced in cells growing in GLY compared to in those in GLU (Fig. 2A). As mentioned above, *pka1-Δ* cells mimic glucose starved cells. Wild-type cells divided at a reduced size on GLY, similar to *Pka1-Δ* cells on GLU (Fig. 2B,C). However, this is not due solely to reduced Pka1p activity, because growth of *pka1-Δ* on GLY further reduced cell size at division (Fig. 2C).

Double mutants of *pdc101-Δ* and *etd1-Δ* with *pka1-Δ* grew at all temperatures on GLU medium (Fig. 2D), suggesting that the rescue of these mutants on GLY is mediated through reduction of Pka1p activity.

We observed that ∼10% of *etd1-Δ* cells at 36°C in GLU or GLY were binucleated and postmitotic (Fig. 2E,F). Thus, the mechanism(s) that compensate for *etd1-Δ* at 36°C are imperfect. At 29°C the fraction of binucleated and multinucleated *etd1-Δ* cells increased in GLU, but not in GLY or in *etd1-Δ pka1-Δ* in GLU (Fig. 2E,F). Therefore, reduced Pka1p activity bypasses the requirement for Etd1p in cytokinesis.

Given that GLY medium and *pka1-Δ* both rescue the cytokinesis defect of *etd1-Δ*, we tested their effects on SIN mutants. GLY medium raised the restrictive temperature of most of the mutants (Table 1). Deletion of *pka1* in SIN mutant backgrounds on GLU revealed a similar pattern of rescue to that in GLY medium (Table 1). We also observed a negative interaction of *pka1-Δ* with *cdc16-116*. These data are consistent with the view that Pka1p is a negative regulator of the SIN. However, neither GLY medium nor *pka1-Δ* bypass the requirement for the SIN. SIN mutants were not rescued by *wee1-6*, indicating that rescue is not due to the small size of *pka1-Δ* cells (data not shown). Likewise, rescue of SIN mutants is not mediated by glycerol acting as a cryoprotectant, given that growth on GLY medium that also contained 3% (w/v) glucose abrogated it (data not shown).

**Table 1.**
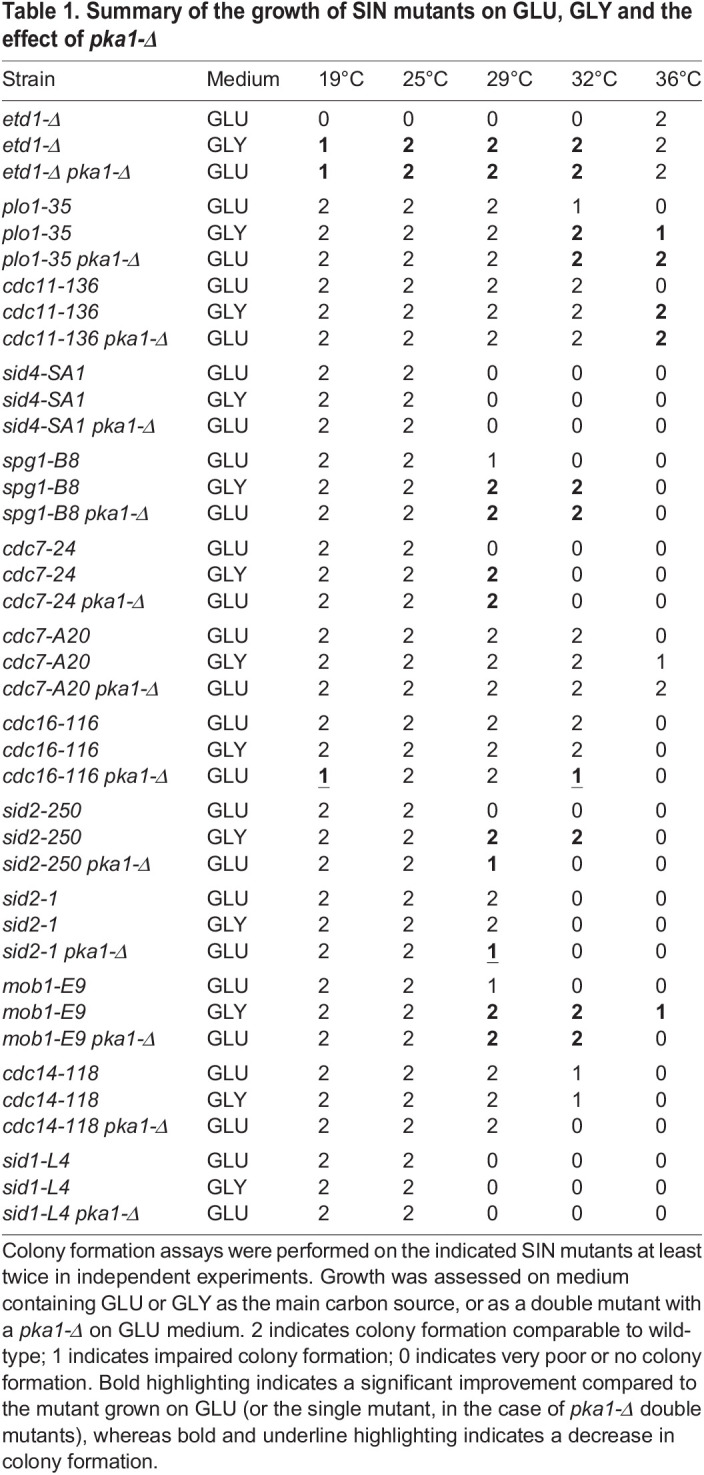
Summary of the growth of SIN mutants on GLU, GLY and the effect of *pka1-Δ*

Genetic evidence indicates that PP2A opposes SIN signalling. The rescue of SIN mutants by *pka1-Δ* and growth on GLY medium is similar to the genetic interactions observed between SIN mutants and *ypa2-Δ* (a regulator of PP2A) or *ppa2-Δ* (the main catalytic subunit of PP2A) (Goyal and Simanis, 2012). The phosphatase regulator Sds23p is activated in low glucose and is required for cell division under these conditions (Deng et al., 2017; Hanyu et al., 2009). Sds23p could increase SIN activity by inhibition of the phosphatases it regulates, which include PP2A, PP1 and PP6. Furthermore, either Pka1p or Sds23p could regulate the SIN inhibitory phosphatase (SIP) complex (Singh et al., 2011). Pka1p might also affect SIN signalling by reducing CDK1 activity (Chang et al., 2001; Dischinger et al., 2008; Guertin et al., 2000; Yamano et al., 1996), given that it affects assembly of the APC/C (Yamashita et al., 1996) and the stability of Cdc25p (Kishimoto and Yamashita, 2000a,b; Tallada et al., 2002). Assembly of the CAR in GLY medium is facilitated by reduced Pka1p activity (Malecki and Bähler, 2016; Prieto-Ruiz et al., 2023), which might also reduce the requirement for SIN signalling in CAR assembly.

SIN protein localization can serve as a proxy for SIN signalling, so we examined whether the distribution of Cdc7p–GFP, GFP–Sid1p and Mob1p–GFP was affected by temperature, carbon source or *pka1-Δ*. We pooled data from the early mitotic cells (LF≤0.4; where LF is the distance of SPB separation as a fraction of cell length) to represent the ‘early SIN’ and from anaphase B cells (LF >0.4) to represent the ‘late SIN’. The distribution of the three proteins in *pka1-Δ* cells or cells grown on GLY was similar to that of wild-type cells growing on GLU (Fig. S3A–G). Nonetheless, there were two notable differences. First, at 36°C, CAR association of Mob1–GFP was more readily detectable in GLY and *pka1-Δ* than in GLU (Fig. S3A). The second was a reduction in GFP–Sid1p signal in *pka1-Δ* cells in GLU at 36°C in both early and late mitosis (Fig. S3B,C). This might indicate a role for Pka1p in GFP–Sid1p maintenance at the SPB at higher temperatures and in regulating association of Mob1p–GFP with the CAR. We do not know whether these differences reflect altered turnover rates of GFP–Sid1p and Mob1p–GFP at the SPB and CAR.

We then examined the effect of carbon source or *pka1-Δ* on localization of these SIN proteins in *etd1-Δ* cells grown in GLU or GLY at 36°C and shifted to 29°C. Mob1p–GFP was detected on both SPBs in *etd1-Δ*, similar to the result in wild-type (Fig. S3D,E). No Mob1p–GFP rings were detected in GLU at 29°C, consistent with cytokinesis failure in *etd1-Δ* (Fig. S3A). However, CAR association of Mob1p–GFP was detected in 10% of *etd1-Δ* cells in GLY and in 14% of *etd1-Δ pka1-Δ* cells in GLU at 29°C, indicating a partial rescue of Mob1p–GFP CAR association (Fig. S3A).

The distribution of Cdc7p–GFP during mitosis in *etd1-Δ* was not altered at 36°C, indicating that Cdc7p–GFP does not require Etd1p for SPB association at 36°C (Fig. S3F,G). In late mitosis, over half the cells did not show a signal at 29°C, consistent with cytokinesis failure (Fig. S3G). This was rescued by *pka1-Δ* or by growth on GLY (Fig. S3F,G). No Cdc7p–GFP was detectable in ∼10% of *etd1-Δ* cells grown on GLU or GLY at 36°C (Fig. S3F,G) consistent with the fraction of *etd1-Δ* cells that are binucleated and postmitotic at 36°C (Fig. 2E,F). Activation of the checkpoint monitoring CAR integrity (Le Goff et al., 1999; Liu et al., 2000) results in the association of Cdc7p–GFP with the SPB of one of the postmitotic nuclei (Liu et al., 2000). However, <5% of the postmitotic cells displayed Cdc7p–GFP on the SPB (data not shown). Thus, although a minority of the binucleated cells might be arrested by the CAR checkpoint, most are not. *S. pombe* can divide as a dikaryon (Okazaki and Niwa, 2008) so some of these binucleated cells might propagate as such.

After 5 h at 29°C, 85% of LF>0.4 *etd1-Δ* cells in GLU medium had no GFP–Sid1p signal, consistent with the failure of cytokinesis (Fig. S3C). Unexpectedly, at 36°C 73% of LF≤0.4 and 64% of LF>0.4 cells also had no detectable GFP–Sid1p signal (Fig. S3B,C). This was partially rescued by *pka1-Δ* on GLU or by growth on GLY. The level of GFP–Sid1p did not change in *etd1-Δ* cells (data not shown). However, *sid1-239* and *cdc7-24* were epistatic to *etd1-Δ* at 36°C, so Cdc7p and Sid1p remain essential for cytokinesis in *etd1-Δ*. It is possible that association of GFP–Sid1p with the SPB is transient and harder to detect in *etd1-Δ* at 36°C because Etd1p is required for the transition from the early to the late SIN.

**Fig. 3.**
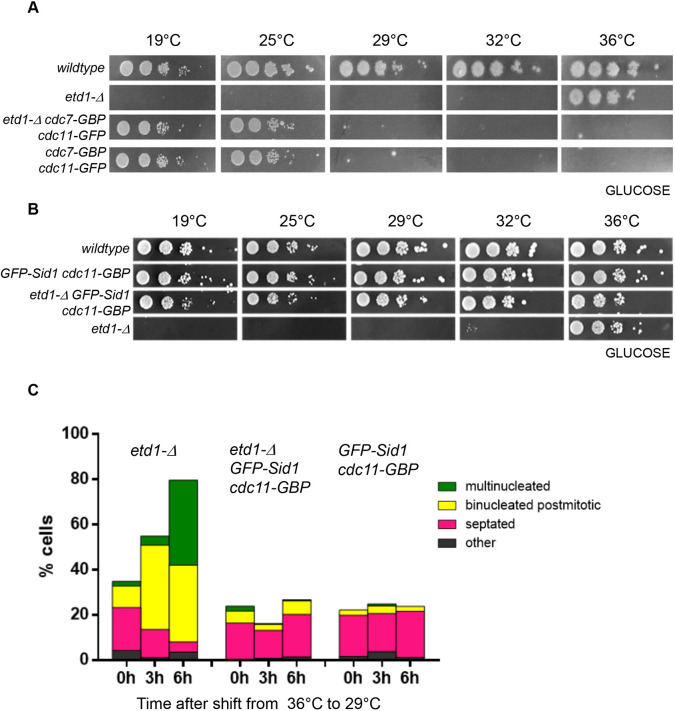
**Rescue of *etd1-Δ* by recruitment of Sid1p or Cdc7p to the SPB.** (A,B) The colony forming ability of cells of the indicated genotypes was assessed at the indicated temperatures. Images in B and C are representative of three repeats. (C) Cells were grown at 36°C and shifted to 29°C for the indicated times. The phenotypes were quantified after fixation and staining with DAPI and Calcofluor. Results are the mean for three repeats with 100 cells analysed per repeat.

The absence of Cdc7p and Sid1p from the SPB in *etd1-Δ* at 29°C on GLU prompted us to test whether tethering these proteins to the SPB using the GFP-GBP system (Rothbauer et al., 2008, 2006) could rescue *etd1-Δ*. We tethered Cdc7p to Cdc11p in wild-type and *etd1-Δ* cells. Colonies formed at 19°C and 25°C (Fig. 3A) indicating rescue of *etd1-Δ*. However, at ≥29°C, *etd1-Δ cdc7-GBP cdc11-GFP* cells could not form colonies (Fig. 3A) and became multiseptated (data not shown), as described previously for *etd1^+^* cells (Chen et al., 2017). In contrast, *GFP-sid1 cdc11-GBP* cells grew on GLU medium at all temperatures (Fig. 3B). Most cells divided normally, though some post-mitotic cells were observed (Fig. 3C). *Etd1-Δ GFP-sid1 cdc11-GBP* cells also grew at all temperatures, whereas *etd1-Δ* could only do so at 36°C (Fig. 3B,C). We conclude that tethering of either Cdc7p or Sid1p to Cdc11p rescues *etd1-Δ*. These data support the idea that both Cdc7p and Sid1p act downstream of Etd1p. SPB tethering of Cdc7p–GFP or GFP–Sid1p might compensate for the impaired transition from the early to the late SIN in *etd1-Δ*. The less dramatic effect of tethering Sid1p to the SPB might be because Sid1p requires activation (directly or indirectly) by Cdc7p.

In summary, our studies reveal a link between the metabolic state of the cell and the activity of the SIN. This is mediated in part via Pka1p, whose activity decreases when cells grow on a non-fermentable carbon source. The mechanism by which Pka1p influences the SIN remains to be elucidated, but we favour a model in which it regulates inhibitors of the SIN, such as PP2A.

## MATERIALS AND METHODS

### Yeast methods

#### Growth

*Schizosaccharomyces pombe* (see below for details of strains) was grown as described (Hagan et al., 2016; Moreno et al., 1991). Unless otherwise stated, cells were grown in complete yeast extract medium [YE; 0.5% (w/v) Difco Bacto Yeast extract with 50 mg/l of adenine, histidine, lysine, leucine and uracil] with either 3% (w/v) glucose (GLU medium) or 3% (v/v) glycerol plus 0.1% (w/v) glucose (GLY medium) as the main carbon source. EMM2 minimal medium was used for plasmid selections with addition of required supplements as required [50 mg/l of adenine (A), histidine (H), lysine (K), leucine (L) or uracil (U)]. Phloxin B (Sigma) was added to the solid media at a concentration of 5 mg/l to identify the colonies containing large number of dead cells (e.g. lysed mutants) or diploids. Cell concentrations were determined by a Neubauer hemocytometer (depth 0.1 mm) or Countess II Automated Cell Counter (Thermo Fisher Scientific) used according to the manufacturer's recommendations.

To perform phenotype analysis for any strain in GLU and GLY medium, a single colony was resuspended in water and equal volumes of cell suspension were inoculated into either GLU or GLY medium at permissive temperature as a starter culture. The next day, cells were inoculated for exponential growth by using these starter cultures to eliminate stress caused by a medium shift.

For colony formation assays, cells were grown to ∼4×10^6^/ml. Serial 10-fold dilutions were made in growth medium such that the densest spot corresponds to ∼2×10^4^–4×10^4^ cells. Plates were incubated at each temperature until the wild-type cells had formed colonies (2–3 days at 36°C, 5–6 days at 19°C).

#### Genetics

Gene names are represented by three italicized lower-case letters and a number, e.g. *etd1*. Replacement of a gene by another is indicated by ‘::’ followed by the name of replaced gene, e.g. *etd1::ura4^+^* (Kohli and Nurse, 1995). For simplicity in the text, gene replacement alleles are denoted by the gene name and the Greek delta letter ‘Δ’ separated by a hyphen, e.g. *etd1-*Δ.

Strains are from lab stocks, or the indicated source (see Table S1). Strains were checked using standard methods. Compound mutant strains were constructed using tetrad dissection from standard lab strains.

For dominance tests, non-sporulating diploids were generated by using the *mat2-P_i_-102* allele in an *h^90^* background (Egel, 1973) and mating to an *h^−^* mutant strain. Auxotrophies were used to counter-select the parental strains.

To test whether *cdc7-24* and *sid1-239* are epistatic to *etd1-Δ*, *etd1::ura4+ leu1-32::int-nmt81-etd1+(leu1+) ura4-D18 ade6-M216 h^−^* was crossed to *cdc7-24 ura4-D18 h^+^* and *sid1-239 ura4-D18 h^+^*. Spores were germinated at 36°C on GLU medium. After 3 days, tetrads were scored visually to determine the fate of spores that did not give rise to colonies. Plates were replicated to GLU at 25°C to identify colonies bearing both the *etd1::ura4^+^ leu1-32::int-nmt81-etd1^+^(leu1^+^)* alleles given that cells become elongated and lyse due to the cold-sensitive phenotype of *etd1::ura4^+^*.

The transition point of *pdc101-r3* (*etr3-1*) cell was calculated by using the formula: X_TP_= 1–[ln(*N*/*N*_0_) /ln(A)] (where *N*=cell density at the termination of growth, *N*_0_= cell density at time of temperature shift and A=the number of daughter cells) as described previously (Howell et al., 1975). This assumes that the inactivation is rapid and complete.

#### Isolation of *pdc101-3* cells

*etd1-M9* cells were grown to 2×10^6^/ml in complete medium at 19°C, harvested, resuspended in water at approximately 10^8^ cells/ml and plated onto GLU medium at 29°C, spreading ∼3×10^7^ cells per plate. The plates were incubated at 29°C for 5 days. Colonies that formed were replica plated to 36°C to identify heat-sensitive cells. These were picked and streaked at 29°C. Colonies were screened by backcrossing to *ura4-D18* and free spore analysis to confirm the presence of an extragenic suppressor. Colonies of interest were analysed by tetrad dissection after crossing to *ura4-D18*. *pdc101-3* (*etr3*) was retained for analysis due to the phenotype of the mutant.

Strains are available on request from the corresponding author, and key strains have been deposited in the National BioResource Project – Yeast in Japan.

### DAPI and Calcofluor staining

To visualize the nucleus and septum, cells were grown to exponential phase, collected by centrifugation (1500 ***g*** for 2 min) and fixed with ice-cold 70% (v/v) ethanol. As described in Moreno et al. (1991), cells were stained in 1× PBS containing 20 µg/ml DAPI (Sigma, cat. no. 28718-90-3) and 30 µg/ml Calcofluor (Sigma, cat. no. 4404-43-7). Images were captured on a Zeiss Axiophot microscope (Zeiss, 100×/1.4 NA lens) using a Nikon D5600 camera and processed using Photoshop 23.2.1 and ImageJ 1.53f51. The cell length of dividing cells (septated cells) was also measured using ImageJ.

### Fluorescence microscopy and analysis of SIN protein localization

The methods described in Wachowicz et al. (2015) were followed, except as indicated below. First, cells were not synchronized by elutriation due to the difficulty in obtaining satisfactory separation of *etd1-Δ*. Second, we noted that *etd1-Δ* showed very strong negative interactions at 36°C with GFP-tagged SIN proteins, if an mCHY-tagged SPB marker was present (data not shown).

We therefore fixed cells with cold methanol after harvesting by filtration, and then stained with DAPI and Calcofluor as described previously (Hagan et al., 2016). Nuclear morphology was used to identify mitotic cells. Only mitotic cells were used for the analysis of SIN protein localization. Exponentially growing cells were harvested by filtration using MF^TM^ Millipore filters (0.45 µm) (HAWP02500) and fixed with 1 ml of −20°C 100% (v/v) methanol. The samples were stored at −20°C until imaging. For visualization, cells were washed once with 1× PBS and stained in 1× PBS containing DAPI and Calcofluor. Images were acquired on Visitron CSU-W1 spinning disk using a Hamammatsu EMMCD camera with U PLAN S APO Mag/NA 100×/1.40 objective lens. Images were taken with a step-size of *z*= 0.3 µm. Maximum intensity projection of the individual *z*-stacks was undertaken with VisiView software.

The length fraction was measured for >100 cells for each independent experiment and the data were pooled for plotting, as described (Wachowicz et al., 2015). The cells were grown in different media types at least twice independently. The number of cells analysed for each SIN protein examined is given in Table S3.

### Molecular biology

#### Oligonucleotides

A list of oligonucleotides used for mutagenesis of *etd1* is provided in Table S2.

#### Generation of *etd1-M9* by low fidelity PCR

The marker recognition system was used to generate and insert mutants into the genome (Tang et al., 2011). Insertion of *etd1* into pHdelCu4+ was undertaken as follows. Primers P3 and P4 were used to amplify the 3′UTR of *etd1*, and the C-terminal fragment of *etd1* was amplified using P1 and P2. Nested PCR was undertaken on these fragments using P1+P4. This was digested with SalI and PvuII and cloned into pHdelCu4+ cut with the same enzymes. This was linearized with SmaI and used to transform *ura4-D18 his5-D1* to uracil prototrophy. This generated the strain *etd1::etd1*-His5delC-(ura4*^+^*). High fidelity PCR was performed using Phusion® High-Fidelity DNA Polymerase enzyme (New England Biolabs, cat. #M0530) according to the manufacturer's instructions. Insertion of *etd1* into pH5C (Tang et al., 2011) was undertaken as follows. PCR was performed on genomic DNA using primers P4 and P5. The product was digested with SalI and PvuII and cloned into pH5C that had been digested with SalI and EcoRV. Mutagenic PCR was performed on this template, using primers P0 and P5 following the protocol of Cadwell and Joyce (2006). The product was used to transform Byr4::byr4-His5delC-(ura4+) to histidine prototrophy. Colonies were allowed to form at 19°C and replicated to 29°C to identify conditional mutants. The *etd1-M9* mutant picks up suppressors frequently. To facilitate its use, we rescued the mutation by inserting *etd1^+^* at *lys1* (Table 1). Compound mutants were made by tetrad dissection and lys+ progeny with the appropriate genotype were analysed promptly.

#### Genome sequencing

Libraries for whole genome sequencing were prepared according to manufacturer's instructions with the TruSeq DNA PCR-free kit (Illumina) starting from 2200 ng of good-quality genomic DNA. Libraries were subsequently loaded at 8 pM on a MiSeq v3 flow cells (Illumina) and sequenced according to manufacturer's instructions, yielding pairs of 300 nt reads (PE300). The mean output was 2×10^6^ reads per sample, corresponding to a coverage of >50×. Reads were trimmed of their adapters with bcl2fastq v2.18 and quality-controlled with fastQC v0.11.5.

Mapping and SNP calling was done with CLC Genomics Workbench 10.0, with Ensembl ASM294v2 annotations. The data have been submitted to the sequence read archive under the submission ID SUB13674639.

#### *S. pombe* transformation

Fission yeast cells were transformed by the lithium acetate method as depicted in Okazaki et al. (1990). For transformations with PCR products generated from tagging vectors, the protocol of Bähler et al. (1998) was used.

#### Generation of a sporulating *pdc101-Δ/pdc101+* diploid

The heterozygous null allele for *pdc101* SPAC1F8.07c was purchased from Bioneer™. The *h^+^*/*h^+^* Bioneer strain was crossed to an *h^−^*/*h^−^ ade6-M210 leu1-32 ura4-D18* strain and sporulating, G418^R^, ade^+^ *ade6-M210*/*ade6-M216* diploids were identified.

### Biochemistry

#### Non-acetylation enzyme-immunoassay for measuring intracellular cAMP level

To measure intracellular cAMP level, yeast cells were grown in different types of media three times independently and collected as described below. The cAMP Biotrak^TM^ EIA (non-acetylation protocol) (Cytiva, GERPN2251) was used. All buffers used were supplied in the kit, and used according to the manufacturer's recommendation. For this purpose, 2×10^8^ exponentially growing cells were collected by centrifugation for 2 min at 1500 ***g*** at room temperature. After washing the cells once washed with cold water, the cell pellet was collected in an Eppendorf tube and immediately immersed in liquid nitrogen and stored at −80°C until the assay procedure. The assay reagents and standards provided with the kit were prepared according to the manufacturer's instructions. The cells were resuspended in 200 µl of lysis reagent 1B provided with the kit and disrupted in a MagNA Lyser (Roche) using Lysing Matrix C beads (MP Biomedicals, 116912100) at 4°C during 2 cycles of 45 s at setting 6.5. The beads were washed with 200 µl of lysis reagent 1B, vortexed and the supernatant was collected. The assay method was performed according to the manufacturer's instructions. Briefly, 100 µl of samples or standard solutions were added into the wells covered with donkey anti-rabbit-IgG and incubated with 100 µl of antiserum (rabbit anti-cAMP antibody) for 2 h at 4°C. Next, 50 µl of cAMP–peroxidase conjugate was added into each well and incubated for 1 h at 4°C. After the incubation, all the solution was aspirated and washed four times with 400 µl of wash buffer. 150 µl of enzyme substrate was dispensed into all wells and mixed on a microplate shaker for 1 h at room temperature. As recommended in the protocol, the reaction was halted by adding 100 µl of 1 M sulfuric acid (Sigma, 339741). The optical density (OD) at 450 nm was determined within 30 min of the addition of 1 M sulfuric acid. The samples were assayed in duplicate for the within-assay precision and mean of the OD measurements were used for plotting. The values obtained for cAMP in wild-type cells were close to the detection limit of the assay, as determined by standard curves. Unfortunately, the results obtained in wild-type cells were not repeatably significantly above background (data not shown). In order to increase the amount of cAMP, the experiment was repeated in a *cgs2-*Δ cell, which lacks phosphodiesterase.

### Statistical methods and sample numbers

A two-tailed unpaired Student's test was performed for the cAMP measurement (Fig. 2A). A one-way ANOVA with the Tukey test for multiple comparisons was performed for the cell length analysis (Fig. 2C). These tests were done using the GraphPad program (v6.0).

For analysis of the cells in different growth media, three biological replicates were performed, and >100 septated cells were measured for each experiment. For the cell length analysis shown in Fig. 2C, the numbers analysed were 1st experiment: *n*=103, 2nd experiment: *n*=135, 3rd experiment: *n*=109.

For the analysis of SIN protein distribution presented in Fig. S3, three biological replicates were performed. The total number of mitotic cells analysed for each condition that are presented in the figure are given in Table S3.

## Supplementary Material



10.1242/joces.261488_sup1 Supplementary information
