# Supplementary material for: A role for the carbon source of the cell and protein kinase A in regulating the S. pombe septation initiation network
Source: J Cell Sci. 2024 Jan 10;137(1):jcs261488. doi: 10.1242/jcs.261488 (PMC10906493; doi:10.1242/jcs.261488)
Supplement: Supplementary information [file joces-137-261488-s1.pdf]

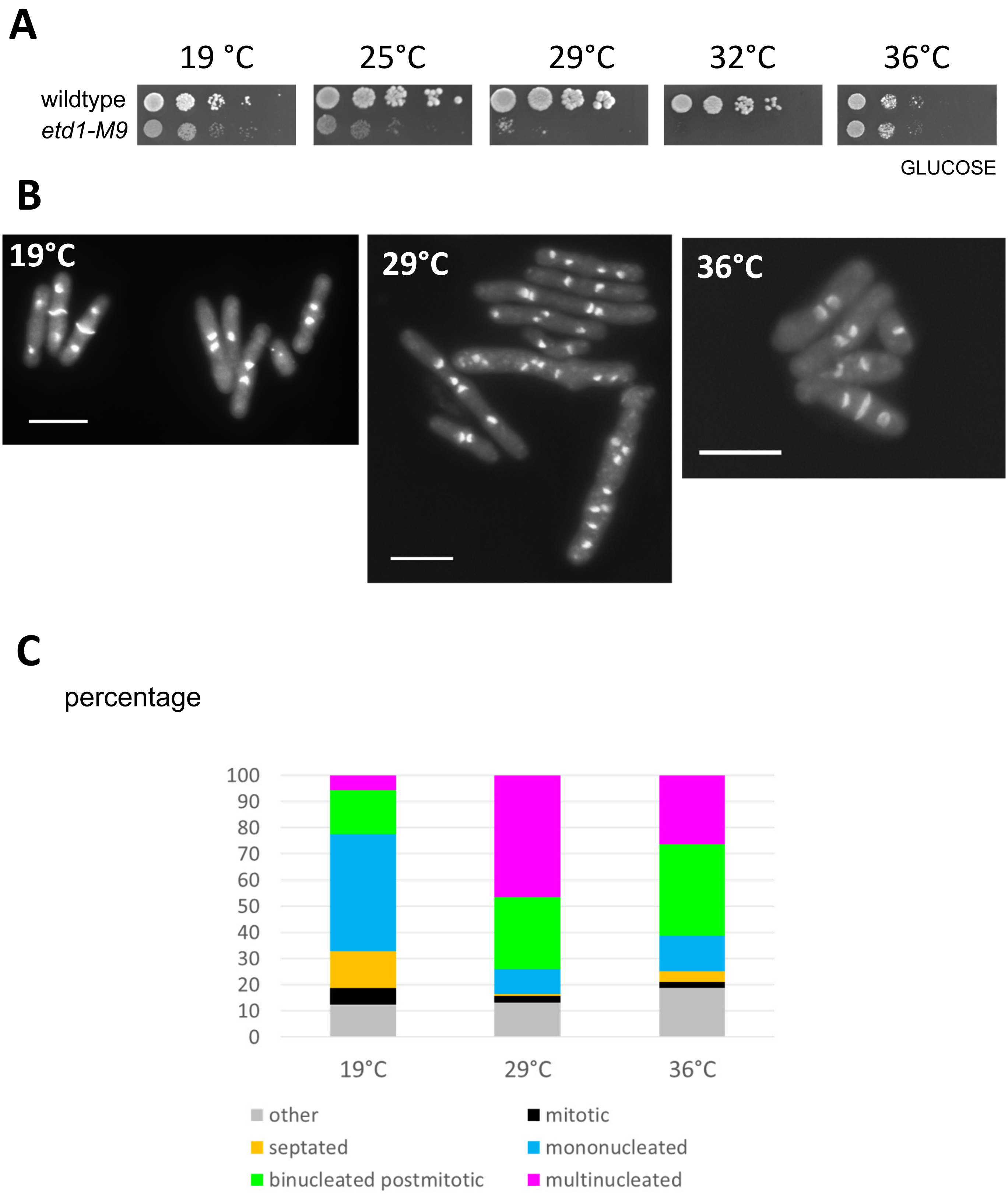

Journal of Cell Science • Supplementary information

Fig. S1. Characterisation of etd1-M9

Panel A:

cells of the indicated genotypes were grown at 19°C and spotted on GLU medium at the indicated temperatures.

Panel B:

etd1-M9 cells were grown to approximately 10^6 ml^{-1} at 19°C. the culture was divided into three parts which were grown at the indicated temperatures for approximately two cycles; 8h (19°C), 6h (29°C) and 5h (36°C). Cells were fixed and stained with DAPI and Calcofluor. The scale bar represents 10 μm.

Panel C:

Quantification of the phenotypes of the cells at the indicated temperatures.

Journal of Cell Science • Supplementary information

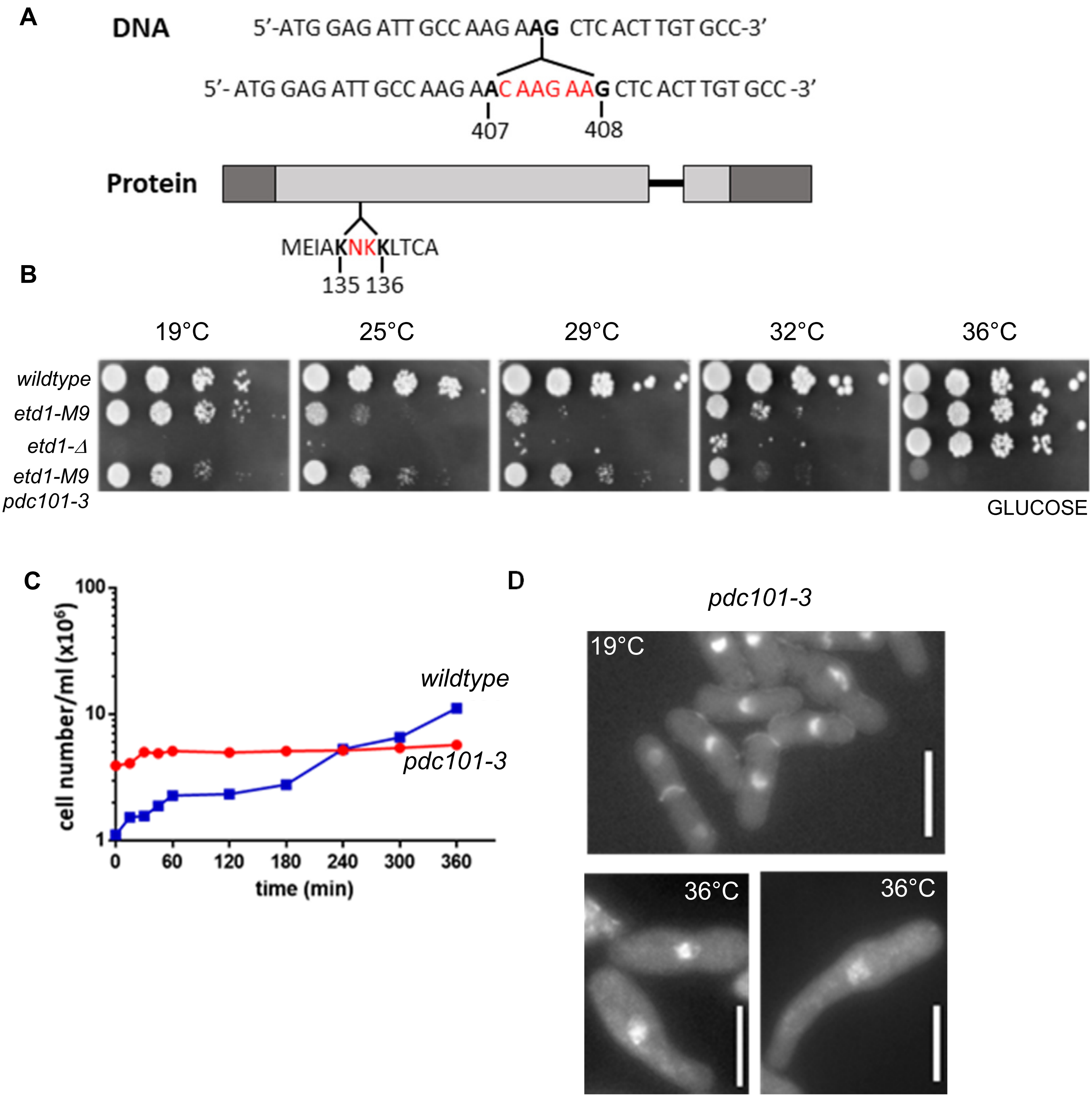

Fig. S2. Characterisation of *pdc101-3*.

Panel A: representation of the mutation in *pdc101-3* and its effect upon the protein sequence. Panel B: strains of the indicated genotype were grown to early exponential phase at 19°C (except *etd1-Δ* which was grown at 36°C). Dilutions were made and spotted at the indicated temperatures. Note that the wildtype and *etd1-Δ* dilution series are the same as those shown in Figure 1A. In Figure 1A, the series corresponding to *etd1-M9* and *etd1-M9 pdc101-3* have been deleted. The images shown in Figure 1A and Figure S2B are derived from images taken from a single plate for each temperature. Panel C: wildtype or *pdc101-3* cells were grown at 19°C and shifted to 36°C. Cell number was determined at the indicated times after shift.

Panel D: *pdc101-3* cells were grown at 19°C and shifted to 36°C for 6h. Cells were fixed and stained with DAPI and Calcofluor. The scale bar represents 10 μm.

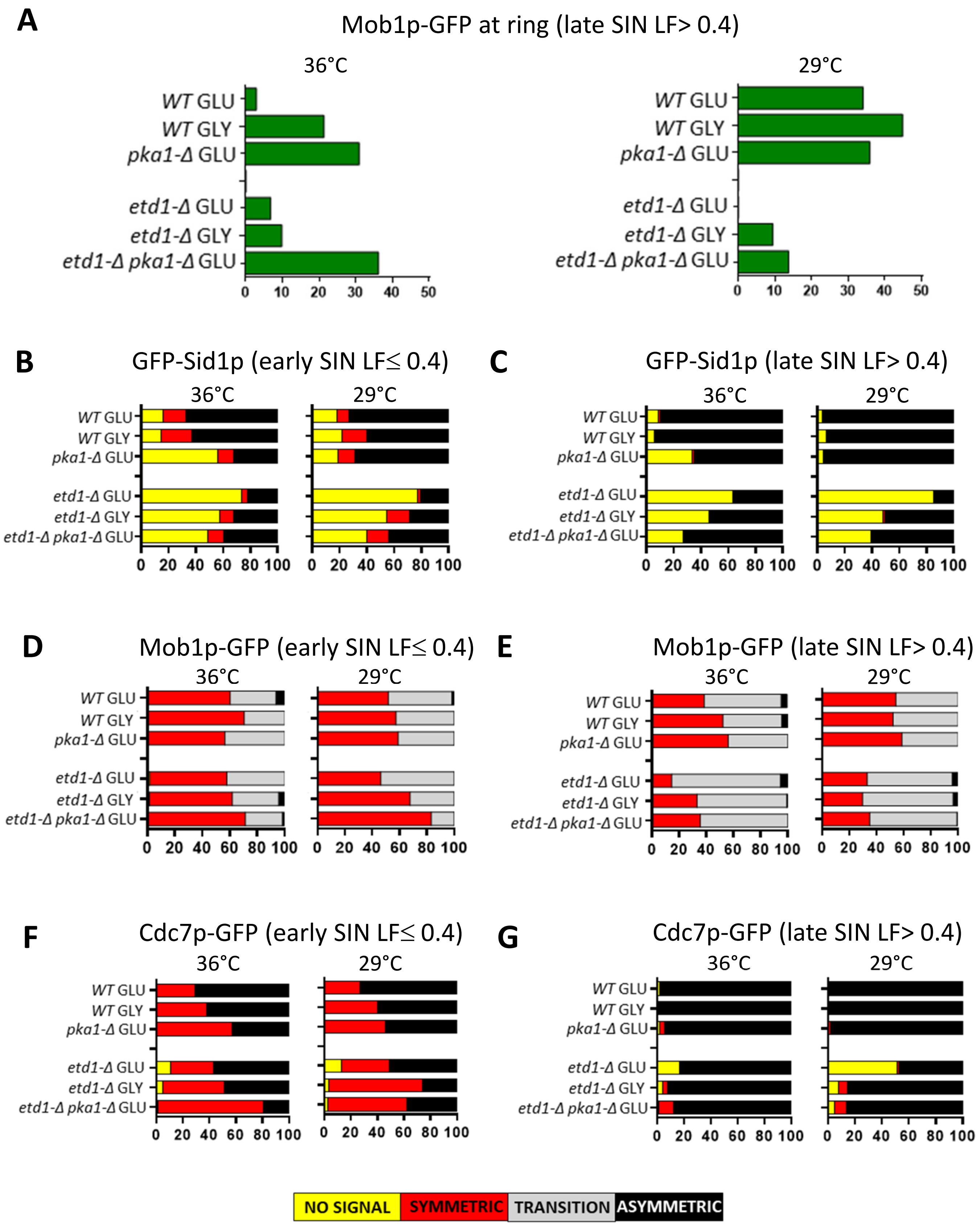

Fig. S3. Analysis of the SPB association of GFP-tagged SIN proteins in GLU and GLY medium, in wildtype and *etd1-Δ* and the effect of *pka1-Δ*.

Wildtype (WT), *etd1-Δ*, *pka1-Δ* or *etd1-Δ pka1-Δ* cells expressing the indicated GFP tagged SIN protein were grown at 36°C to exponential phase in the indicated medium. A fraction of the culture was shifted to 29°C for 5h. Cells were fixed and stained with DAPI. The separation of the nuclei was expressed as a fraction of the cell length. The colour scheme used is defined in (Wachowicz et al.,

Journal of Cell Science • Supplementary information

2015). The measurements of two independent experiments with >100 cells for each were pooled and plotted. Only mitotic cells were used for measurement.

Panel A: Mob1p-GFP association with the CAR in late anaphase cells.

The abscissa of the bar graph represents the percentage of cells that shown Mob1p-GFP associated with the contractile ring. The temperatures and genotypes of the cells analysed is indicated.

Panel B: GFP-Sid1p distribution in cells of length fraction ≤0.4 (early configuration of the SIN)

Panel C: GFP-Sid1p distribution in cells of length fraction >0.4 (late configuration of the SIN)

Panel D: Mob1p-GFP distribution in cells of length fraction ≤0.4 (early configuration of the SIN)

Panel E: Mob1p-GFP distribution in cells of length fraction >0.4 (late configuration of the SIN)

Panel F: Cdc7p-GFP distribution in cells of length fraction ≤0.4 (early configuration of the SIN)

Panel G: Cdc7p-GFP distribution in cells of length fraction >0.4 (late configuration of the SIN)

The abscissa of the stacked bar graphs represents the percentage of cells.

Journal of Cell Science • Supplementary information

Table S1. list of strains used in this study.

972 h-	lab collection (originally Nurse lab)	(Nurse et al., 1976)
leu1-32 h-	lab collection (originally Nurse lab)	(Nurse et al., 1976)
ura4-D18 h-	lab collection (originally Kohli lab, Bern)	(Grimm et al., 1988)
cdc16-116 h-	Lab collection (originally Nurse lab)	(Minet et al., 1979)
sid4-SA1 h-	Lab collection (from Gould lab))	(Chang and Gould, 2000)
cdc11-136 h-	lab collection (originally Nurse lab)	(Nurse et al., 1976)
cdc11-123 h-	lab collection (originally Nurse lab)	(Nurse et al., 1976)
spg1-B8 h-	Lab collection	(Schmidt et al., 1997)
cdc7-A20 h-	Lab collection	(Fankhauser and Simanis, 1994)
cdc7-24 h-	lab collection (originally Nurse lab)	(Nurse et al., 1976)
sid1-L4 h-	V.Simanis unpublished from the screen given in the reference	(Fournier et al., 2001)
sid1-125 h-	Lab Collection (Gould lab)	(Guertin et al., 2000)
cdc14-118 h-	lab collection (originally Nurse lab)	(Nurse et al., 1976)
sid2-1 h-	lab collection	(Fournier et al., 2001)
sid2-250 h-	Lab collection (originally McCollum lab)	(Sparks et al., 1999)
mob1-R4 h-	Lab collection	(Salimova et al., 2000)
plo1-35 h-	Lab collection (originally Hagan lab)	(Anderson et al., 2002)
cdc7-GFP(KAN) ura4-D18 h-	Lab collection	E. Salimova and A.Krapp
GFP-sid1(ura4+) ura4-D18 h-	lab collection (McCollum lab)	(Guertin et al., 2000)
cdc11-GBP(KAN) ura4-D18 h-	Lab collection	
mob1-GFP(KAN) ura4-D18 h-	Lab collection	(Salimova et al., 2000)
ppc89-mCHY(NAT) ura4-D18 h-	Lab collection (original Jaspersen lab)	(Bestul et al., 2017)
aur1::mCHY-atb2(AUR) ura4-D18 h-	Lab collection (original Toda lab)	(Fong et al., 2010)
hht1-RFP(KAN) ura4-D18 h-	Lab collection (original Cooper lab)	(Tomita and Cooper, 2007)
lys1::GFP-atb2(ura4+) ura4-D18 h-	Lab collection	A.Krapp unpublished
pdc101-3 lys1::GFP-atb2(ura4+) ura4-D18 hht1-RFP(KAN) h-	This study	
etd1-M9(ura4+ his5+) ura4-D18 h-	This study	
etd1::ura4+ ura4-D18 leu1-32	Lab collection (original Moreno	(Daga et al., 2005)

ade6-M216 h-	lab)	
etd1::ura4+ leu1-32::int-nmt81-etd1+(leu1+) ura4-D18 ade6-M216 h-	Lab collection	(Daga et al., 2005)
etd1::ura4+ lys1::etd1+ura4-D18 leu1-32	Lab collection	
etd1-M9(ura4+his5+) lys1::etd1+ ura4-D18 leu1-32	Lab collection	
etd1-M9(ura4+his5+) pdc101-3 ura4-D18 h+	This study	
etd1::ura4+ pdc101-3 ura4-D18 h-	This study	
pdc101 ::KAN/pdc101+ ade6-M210/ade6-M216 leu1-32/leu1-32 ura4-D18/ura4-D18 h+/h+	Bioneer S. pombe Genome-wide Deletion Mutant Library	
pdc101 ::KAN/pdc101+ ade6-M210/ade6-M216 leu1-32/leu1-32 ura4-D18/ura4-D18 h+/h-	This study	
pdc101 ::KAN h+	This study	
isp3 ::KAN ura4-D18 leu1-32 ade6-M216 h+	Bioneer S. pombe viable null mutant strain collection (V5)	
etd1::ura4+ pdc101 ::KAN ura4-D18 leu1-32 h-	This study	
pdc101-3 cdc11-136 ura4-D18 h+	This study	
pdc201::KAN ura4-D18 leu1-32 ade6-M216 h+	Bioneer S. pombe viable null mutant strain collection (V5)	
pdc102::KAN ade6-M210 ura4-D18 leu1-32 h+	Bioneer S. pombe viable null mutant strain collection (V5)	
pdc101-3 pdc102::KAN ura4-D18 h-	This study	
pdc101::KAN pdc102::KAN ade6-M210 ura4-D18 leu1-32 h-	This study	
pdc202::KAN ade6-M216 ura4-D18 leu1-32 h+	Bioneer S. pombe viable null mutant strain collection (V5)	
pdc101-3 pdc202::KAN ura4-D18 h-	This study	
pdc101 ::KAN ura4-D18 ade6-M210 leu1-32 h- pDW232(ura4+)-EMPTY	This study	
pdc101 ::KAN ura4-D18 ade6-M210 leu1-32 h- pDW232(ura4+)-pdc102+	This study	
pdc101 ::KAN ura4-D18 ade6-M210 leu1-32 h- pDW232(ura4+)-pdc201+	This study	
wee1-6 h-	lab collection (originally Nurse lab)	(Thuriaux et al., 1980)

Journal of Cell Science • Supplementary information

cdc25-22 h-	lab collection (originally Nurse lab)	(Thuriaux et al., 1980)
pka1::NAT h-	Japanese National BioResource Project	
cyr1: ura4+ ura4-D18 leu1-32 h-	Japanese National BioResource Project	
cyr1::ura4+ pka1-GFP(KAN) ura4-D18 leu1-32 h-	Japanese National BioResource Project	
cyr1 ::KAN ura4-D18 leu1-32 ade6-M210 h-	Japanese National BioResource Project	
pdc101 ::KAN cyr1: ura4+ ura4-D18 leu1-32 h-	This study	
pdc101 ::KAN pka1 ::NAT	This study	
etd1 :: ura4+ cyr1 ::KAN ura4-D18 leu1-32 h+	This study	
etd1 :: ura4+ pka1::KAN ura4-D18 h-	This study	
cdc16-116 pka1::NAT ura4-D18 h-	This study	
cdc11-136 pka1::NAT h+	This study	
cdc7-24 pka1::NAT h-	This study	
spg1-B8 pka1::NAT h+	This study	
mob1-R4 pka1::NAT ura4-D18 h-	This study	
sid2-250 pka1::NAT ura4-D18 h+	This study	
sid4-SA1 pka1::NAT h-	This study	
plo1-35 pka1::NAT h+	This study	
cdc7-A20 pka1::NAT h-	This study	
cdc11-119 pka1::NAT h+	This study	
sid1-L4 pka1::NAT h-	This study	
cdc14-118 pka1::NAT h-	This study	
pka1::NAT ppa2::ura4+ ura4-D18 leu1-32 h-	This study	
ssp2::KAN pka1::NAT ppa2::ura4+ ura4-D18 h+	This study	
spg1-B8 ssp2::KAN pka1::NAT ppa2::ura4+ ura4-D18 h-	This study	
spg1-B8 pka1::NAT ppa2::ura4+ ura4-D18 h-	This study	
etd1::ura4+ cdc7-GFP(KAN) ura4-D18 h+	This study	
etd1::ura4+ GFP-sid1(ura4+) ura4-D18 h-	This study	
etd1::ura4+ mob1-GFP(KAN) ura4-D18 h+	This study	

Journal of Cell Science • Supplementary information

etd1::ura4+ cdc7-GFP(KAN) pka1::NAT ura4-D18 h-	This study	
etd1:: ura4+ GFP-sid1(ura4+) pka1 ::NAT ura4-D18 h-	This study	
etd1:: ura4+ mob1-GFP(KAN) pka1 ::NAT ura4-D18 h-	This study	
pka1 ::NAT cdc7-GFP(KAN) ura4-D18 h-	This study	
pka1 ::NAT GFP-sid1(ura4+) ura4-D18 h-	This study	
pka1 ::NAT mob1-GFP(KAN) ura4-D18 h-	This study	
GFP-sid1(ura4+) cdc11-GBP(KAN) ura4-D18 h-	This study	
cdc11-GBP(KAN) leu1-32 ::GFP-cdc14(leu1+) cdc14+ ura4-D18 h-	This study	
etd1:: ura4+ GFP-sid1(ura4+) cdc11-GBP(KAN) ura4-D18 h-	This study	
his5-D1 ura4-D18 h-	lab collection (originally Balasubramanian lab)	(Tang et al., 2011)
etd1::etd1-His5delC-(ura4^{+})	This study: transformation of his5-D1 ura4-D18	

Table S2. list of oligonucleotides used in this study

Name	Sequence (5’ – 3’)	Purpose
P0	GACGAAGCTCTTTCTAGAAGCGTAGT	mutagenesis of etd1
P1	CAGCTGTCGACTGGAATGGGAAGTATC	mutagenesis of etd1
P2	CGTTATTCCCGGGCACTAAATAGCAAAATC	mutagenesis of etd1
P3	CATTTTAGTGCCCGGGAATAACGATTCATCGG	mutagenesis of etd1
P4	CAGCTGTCGACAATGGATTAAATAGCC	mutagenesis of etd1

Journal of Cell Science • Supplementary information

Table S3. Numbers of mitotic cells analysed for figure S3

	strain	WILDTYPE	WILDTYPE	pka1-Δ	WILDTYPE	WILDTYPE	pka1-Δ	etd1-Δ	etd1-Δ	etd1-Δ pka1-Δ	etd1-Δ	etd1-Δ	etd1-Δ pka1-Δ
	Temperature	36°C	36°C	36°C	29°C	29°C	29°C	36°C	36°C	36°C	29°C	29°C	29°C
	Media	GLU	GLY	GLU	GLU	GLY	GLU	GLU	GLY	GLU	GLU	GLY	GLU
MARKER													
cdc7-GFP		202	208	208	203	209	214	201	202	205	244	212	210
GFP-sid1		211	204	202	220	209	203	207	201	196	201	201	200
mob1-GFP		202	204	201	204	202	201	203	201	204	207	203	206

Journal of Cell Science • Supplementary information

Anderson, M., Ng, S. S., Marchesi, V., MacIver, F. H., Stevens, F. E., Riddell, T., Glover, D. M., Hagan, I. M. and McInerny, C. J. (2002). Plo1(+) regulates gene transcription at the M-G(1) interval during the fission yeast mitotic cell cycle. *Embo J* **21**, 5745-55.

Bestul, A. J., Yu, Z., Unruh, J. R. and Jaspersen, S. L. (2017). Molecular model of fission yeast centrosome assembly determined by superresolution imaging. *The Journal of cell biology* **216**, 2409-2424.

Chang, L. and Gould, K. L. (2000). Sid4p is required to localize components of the septation initiation pathway to the spindle pole body in fission yeast. *Proceedings of the National Academy of Sciences of the United States of America* **97**, 5249-54.

Daga, R. R., Lahoz, A., Munoz, M. J., Moreno, S. and Jimenez, J. (2005). Etd1p is a novel protein that links the SIN cascade with cytokinesis. *Embo J* **24**, 2436-46.

Fankhauser, C. and Simanis, V. (1994). The cdc7 protein kinase is a dosage dependent regulator of septum formation in fission yeast. *Embo J* **13**, 3011-9.

Fong, C. S., Sato, M. and Toda, T. (2010). Fission yeast Pcp1 links polo kinase-mediated mitotic entry to gamma-tubulin-dependent spindle formation. *Embo J* **29**, 120-30.

Fournier, N., Cerutti, L., Beltraminelli, N., Salimova, E. and Simanis, V. (2001). Bypass of the requirement for cdc16p GAP function in Schizosaccharomyces pombe by mutation of the septation initiation network genes. *Arch Microbiol* **175**, 62-9.

Grimm, C., Kohli, J., Murray, J. and Maundrell, K. (1988). Genetic engineering of Schizosaccharomyces pombe: a system for gene disruption and replacement using the ura4 gene as a selectable marker. *Mol Gen Genet* **215**, 81-6.

Guertin, D. A., Chang, L., Irshad, F., Gould, K. L. and McCollum, D. (2000). The role of the sid1p kinase and cdc14p in regulating the onset of cytokinesis in fission yeast. *Embo J* **19**, 1803-15.

Minet, M., Nurse, P., Thuriaux, P. and Mitchison, J. M. (1979). Uncontrolled septation in a cell division cycle mutant of the fission yeast Schizosaccharomyces pombe. *J Bacteriol* **137**, 440-6.

Nurse, P., Thuriaux, P. and Nasmyth, K. (1976). Genetic control of the cell division cycle in the fission yeast Schizosaccharomyces pombe. *Mol Gen Genet* **146**, 167-78.

Salimova, E., Sohrmann, M., Fournier, N. and Simanis, V. (2000). The S. pombe orthologue of the S. cerevisiae mob1 gene is essential and functions in signalling the onset of septum formation. *Journal of cell science* **113**, 1695-704.

Schmidt, S., Sohrmann, M., Hofmann, K., Woollard, A. and Simanis, V. (1997). The Spg1p GTPase is an essential, dosage-dependent inducer of septum formation in Schizosaccharomyces pombe. *Genes Dev* **11**, 1519-34.

Sparks, C. A., Morphew, M. and McCollum, D. (1999). Sid2p, a spindle pole body kinase that regulates the onset of cytokinesis. *The Journal of cell biology* **146**, 777-90.

Tang, X., Huang, J., Padmanabhan, A., Bakka, K., Bao, Y., Tan, B. Y., Cande, W. Z. and Balasubramanian, M. K. (2011). Marker reconstitution mutagenesis: a simple and efficient reverse genetic approach. *Yeast* **28**, 205-12.

Thuriaux, P., Sipiczki, M. and Fantes, P. A. (1980). Genetical analysis of a sterile mutant by protoplast fusion in the fission yeast Schizosaccharomyces pombe. *J Gen Microbiol* **116**, 525-8.

Tomita, K. and Cooper, J. P. (2007). The telomere bouquet controls the meiotic spindle. *Cell* **130**, 113-26.

Journal of Cell Science • Supplementary information
