# Supplementary material for: A role for the carbon source of the cell and protein kinase A in regulating the S. pombe septation initiation network
Source: J Cell Sci. 2024 Jan 10;137(1):jcs261488. doi: 10.1242/jcs.261488 (PMC10906493; doi:10.1242/jcs.261488)
Supplement: Supplementary file 1 [file joces-137-261488_review_history.pdf]

A role for the carbon source of the cell and protein kinase A in regulating the *S. pombe* septation initiation network

Özge Uysal Özdemir, Andrea Krapp, Bastien Mangeat, Marc Spaltenstein and Viesturs Simanis

DOI: 10.1242/jcs.261488

Editor: Renata Basto

Review timeline

Original submission: 13 July 2023
Editorial decision: 9 August 2023
First revision received: 27 October 2023
Accepted: 24 November 2023

Original submission

First decision letter

MS ID#: JOCES/2023/261488

MS TITLE: A suppressor of loss of etd1 function reveals a role for Protein kinase A in regulation of the *S. pombe* Septation Initiation Network.

AUTHORS: Ozge Uysal Ozdemir, Andrea Krapp, Bastien Mangeat, Marc Spaltenstein, and Viesturs Simanis
ARTICLE TYPE: Research Article

We have now reached a decision on the above manuscript.

To see the reviewers' reports and a copy of this decision letter, please go to: https://submit-jcs.biologists.org and click on the 'Manuscripts with Decisions' queue in the Author Area. (Corresponding author only has access to reviews.)

As you will see, the reviewers gave favourable reports but raised some critical points that will require amendments to your manuscript. I hope that you will be able to carry these out because I would like to be able to accept your paper, depending on further comments from reviewers.

Please ensure that you clearly highlight all changes made in the revised manuscript. Please avoid using 'Tracked changes' in Word files as these are lost in PDF conversion.

I should be grateful if you would also provide a point-by-point response detailing how you have dealt with the points raised by the reviewers in the 'Response to Reviewers' box. Please attend to all of the reviewers' comments. If you do not agree with any of their criticisms or suggestions please explain clearly why this is so.

Reviewer 1

Advance summary and potential significance to field

The fission yeast SIN represents a remarkably well characterised signal transduction cascade whose spatial control sits at the heart of the regulation of cytokinesis in fission yeast and, by virtue of the

© 2023. Published by The Company of Biologists under the terms of the Creative Commons Attribution License (https://creativecommons.org/licenses/by/4.0/).

conservation of function in budding yeast and neurospora is assumed to do so throughout fungi. As such, extending insight into the SIN regulatory network will be of interest to the broad readership of Journal of Cell Science. Etd1 is one of the least understood components of the SIN and so this manuscript is addressing a key missing piece of the SIN jigsaw. Through a pathway of genetic analyses Ozdemir et al show how Etd1 function is tied in to carbon metabolism and reveals a major contribution of cAMP signalling to SIN control. The conclusions are consistent with the data and I am supportive of publication.

Comments for the author

My one comment that the authors may wish to consider is that I found it extremely hard to follow the experiments in figure 6 as the analysis is so detailed and yet the key points are summarised succinctly in the discussion around lines 457 where is states “We observed that a significant fraction of etd1-Δ cells had no detectable GFP-sid1p at the SPB, irrespective of the growth medium or presence of pka1-Δ. Since loss of sid1 (or cdc7) function is epistatic to etd1-Δ at 36°C, we do not think that these cells are dividing without cdc7 or sid1 function.”

Perhaps the key data in figure 6 could be extracted and the rest presented as supplementary information?
Also, there is an entire dataset on Mob1-GFP on the SPB in Figure 6e and F and yet no reference to it in the section between lines 342 and 348 that refers to the Mob1-GFP on the ring rather than the SPB and so this section needs re-writing or, more simply a broader summary with reference either to supplementary information or data not shown.

These issues apart, the phenotypic description of the link between carbon metabolic status, Etd1 function and the SIN are clear and make an important contribution to the expanding literature on this important fungal regulatory network.

Minor points:
Line 203&204 pcd101-3 – this is confusing as it is referring to a figure where pdc101-3 is still referred to as etr3-1 Line 277 Figure 3A,B – is this not Figure 5A,B?
Line 296 – presumably the definition of LF is for binucleate cells only?
Line 319 Pka1p “…..may play in the role in the transition from the….’ > “…..may play a role in the transition from the….’
Line 340 and 341 6F – is it not 6G Line 365 (figure 5A, 5B) > (figure 6A,6B)
Line 409 to 412 – needs re-writing

<u>Reviewer 2</u>

Advance summary and potential significance to field

This paper reports the isolation of a temperature-sensitive mutation in etd1, a regulator of the septation initiation network in the fission yeast Schizosaccharomyces pombe. Characterization of this mutant leads to the finding that the cAMP pathway via Pka1 modulates the SIN as a function of carbon source.
Careful examination of SIN component localization suggests that Pka1 inhibits a step of SIN activation during anaphase, perhaps via the APC/C. These are interesting findings since the Pka1 pathway and carbon source have not previously been described as modulators of SIN function. Still, the exact mechanism by which Pka1 intersects with the SIN has not been determined. The logic of the work and the data are clear. However, the paper suffers from a careless and somewhat disorganized presentation. If found suitable for JCS, I recommend condensing to a report format of 4 main display items and 4 supplementary items, particularly because no significant new information on etd1 function is presented and figures that essentially recapitulate previously published data could be placed in supplement. The main finding here is that cAMP levels and Pka1 modulate SIN function in a carbon source-dependent manner by an unknown mechanism and these data can be presented in 4 figures.

Comments for the author

Major comments:

© 2023. Published by The Company of Biologists under the terms of the Creative Commons Attribution License (https://creativecommons.org/licenses/by/4.0/).

1. The manuscript deserves another round of thorough editing.

- As a first example, there is a word missing from the second sentence of the abstract and an extra word in line 42.
- Word capitalization and use are inconsistent. Figure, figure; HIPPO Hippo; Glucose, glucose; D, Δ; etc.
- The use of commas should be re-examined to improve readability.
- In the instance of multiple references in the text, they should be in the same format.
- Considering references, there are some odd choices and omissions. As an example, Chan et al., 2017 is not an appropriate reference for the statement in the text that it is supposed to reference. Please re-examine referencing throughout.
- Labeling of figures should use nomenclature rules – lowercase italic for strains. wt or WT or wildtype for consistency.
- The introduction is too long. Some of the material on metabolism would be better suited to the results section where it would, in doses, set up the experimental design. There is also repetition as it stands.
- Results are presented in figure legends (e.g. 2B, 4A, 4C) and should be instead only in the results section.
- Line 738: compared
- Line 243. The sentence beginning in this line should be re-worded. It is clear what message is trying to be conveyed but the wording is not quite correct.

It is not known that the indicated mutant proteins are denatured at non-permissive temperature and this is what is predicted here.

- The paragraph lines256-161 set up Figure 6, not Figure 5; it is in the wrong place in the manuscript. In general, the organization of what appears where needs to be scrutinized to remove redundancy and place information where it should be.
- The data showing images of Sid1 localization is missing. Line 365 refers the reader to Figure 5 but that does not show Sid1 localization. Sid1 localization is summarized in Figure 6, C and D and behaves as expected given the localization of the other components.
- Methods should be in methods. Some details are lacking. Spot assays as example – what dilutions? Tagging vectors were not used for transformation; PCR products were used. Etc.

2. Figure 5 and 7B: I am unfamiliar with the definition of “compartmented”. Does MN mean >2? Figure 5 and 7B would benefit from diagrams of what cells are being counted as what.

Minor comments:

1. Scale bars are needed in Figure 1B and in 1C, it is not defined in the legend.
Something is masking part of two images in Figure 1C. The quality of the most right-hand image in Figure 1C could be improved. Importantly, the conclusions would be bolstered by some quantitative data.

2. A significant fraction of the world’s population is red-green colorblind so to make the work more inclusive, I recommend using a magenta-green rather than red-green false color combination so that everyone can appreciate the images.

First revision

Author response to reviewers' comments

Replies to referees

The authors are grateful to the referees for their constructive and positive comments about the paper. The referees draw attention to the fact that there are many typographical errors as well as some mis-citations of primary literature. As senior author, I wish to apologise for the substandard presentation of the draft paper. I thank them for their patience and generosity in allowing the paper to be revised.

© 2023. Published by The Company of Biologists under the terms of the Creative Commons Attribution License (https://creativecommons.org/licenses/by/4.0/).

Referee 2 suggested that the paper should be condensed to a short report, which was also proposed by the editor. We have therefore reorganised the paper substantially. In some instances, a point-by-point reply to some of the comments below is no longer relevant (figure numbers, for example). We have retained the majority of the data from the original paper. It has been reorganised as described below. **The figure numbers in the subheadings below refer to the figures in the initial submitted version.**

Figure 1:
As suggested by the referees, we have transferred the preliminary analysis of *etd1-M9* from the main body (figure 1) to supplemental data, now **figure S1**. We have replaced the preliminary analysis of Cdc7p-GFP localisation with a presentation of the DAPI-Calcofluor phenotype of the mutant at 19°C, 29°C, and 36°C. We have not been able to undertake a full quantitative analysis of this mutant with regard to SIN protein localisation because all the authors of the paper have now left the lab, which will soon close due to my retirement. Dr Krapp generated the DAPI images, but neither of us had the time to make the *etd1-M9* SIN-GFP strains and perform the quantitative analysis. I apologise for this. If the referees or the editor wish me to restore the data that was previously in panel 1C, I shall be happy to do so.

Figure 2:
The description of *etr3-1* now becomes **figure S2 panels A, C and D**. We have also included a spot growth assay showing how *etr3-1/pdc101-3* rescues *etd1-M9*, which is how the mutant was identified (panel B). One of the referees also pointed out the potential for confusion as the change nomenclature changes from *etr3-1* to *pdc101-3*. We agree, and have resolved it by altering the text in the revised version to discuss the sequencing and allelism with *pdc101* before any reference to a figure. Thus, the name *pdc101-3* is used throughout. I hope that the solution is considered acceptable.

Figure 3:
This becomes **figure 1** in the revised version. Panel D, complementation of *pdc101-Δ* by *pdc201* overexpression has been removed from the paper due to space restrictions. Though the data are solid, they do not add anything to the core message of the paper.

Figures 4 and 5:
The data are combined and becomes the new **figure** 2. We have also eliminated non-standard abbreviations in the legend to the graph.

Table 1:
No change.

Figure 6:
This becomes the new **figure S3**. We have discussed just the salient points of interest from the data in the text, and minimised the overly long discussion of what was expected. We have also taken care to make sure that all the panels are cited in the text. We thank the referees for their suggestion regarding this part of the data.

Figure 7:
This becomes the new **figure** 3. We have also eliminated non-standard abbreviations in the legend to the graph.

Our replies to the specific comments of the referees are given below (in italics).

Reviewer 1 Advance Summary and Potential Significance to Field:
The fission yeast SIN represents a remarkably well characterised signal transduction cascade whose spatial control sits at the heart of the regulation of cytokinesis in fission yeast and, by virtue of the conservation of function in budding yeast and neurospora is assumed to do so throughout fungi. As such, extending insight into the SIN regulatory network will be of interest to the broad readership of Journal of Cell Science. Etd1 is one of the least understood components of the SIN and so this manuscript is addressing a key missing piece of the SIN jigsaw. Through a pathway of genetic analyses Ozdemir et al show how Etd1 function is tied in to carbon metabolism and reveals a major contribution of cAMP signalling to SIN control. The

© 2023. Published by The Company of Biologists under the terms of the Creative Commons Attribution License (https://creativecommons.org/licenses/by/4.0/).

conclusions are consistent with the data and I am supportive of publication.

Reviewer 1 Comments for the Author:
My one comment that the authors may wish to consider is that I found it extremely hard to follow the experiments in figure 6 as the analysis is so detailed and yet the key points are summarised succinctly in the discussion around lines 457 where is states
"We observed that a significant fraction of etd1-Δ cells had no detectable GFP-sid1p at the SPB, irrespective of the growth medium or presence of pka1-Δ. Since loss of sid1 (or cdc7) function is epistatic to etd1-Δ at 36°C, we do not think that these cells are dividing without cdc7 or sid1 function."

Perhaps the key data in figure 6 could be extracted and the rest presented as supplementary information? Also, there is an entire dataset on Mob1-GFP on the SPB in Figure 6e and F and yet no reference to it in the section between lines 342 and 348 that refers to the Mob1-GFP on the ring rather than the SPB and so this section needs re-writing or, more simply a broader summary with reference either to supplementary information or data not shown.

> ***I agree that this section was very hard to read. I have reorganised the text substantially. I hope that the revised version makes the relevant points more clearly.***

These issues apart, the phenotypic description of the link between carbon metabolic status, Etd1 function and the SIN are clear and make an important contribution to the expanding literature on this important fungal regulatory network.

Minor points:
Line 203&204 pcd101-3 – this is confusing as it is referring to a figure where pdc101-3 is still referred to as etr3-1 ***I have dealt with this as described above***
Line 277 Figure 3A,B – is this not Figure 5A,B? ***Correct - I apologise for the error***
Line 296 – presumably the definition of LF is for binucleate cells only? ***Correct.***
Line 319 Pka1p "…..may play in the role in the transition from the….' > "…..may play a role in the transition from ***This section has been completely rewritten.***
the….'
Line 340 and 341 6F – is it not 6G ***Correct - I apologise for the error***
Line 365 (figure 5A, 5B) > (figure 6A,6B) ***Correct - I apologise for the error***
Line 409 to 412 – needs re-writing ***this section has been completely rewritten.***

*****
Reviewer 2 Advance Summary and Potential Significance to Field:
This paper reports the isolation of a temperature-sensitive mutation in etd1, a regulator of the septation initiation network in the fission yeast Schizosaccharomyces pombe. Characterization of this mutant leads to the finding that the cAMP pathway via Pka1 modulates the SIN as a function of carbon source. Careful examination of SIN component localization suggests that Pka1 inhibits a step of SIN activation during anaphase, perhaps via the APC/C. These are interesting findings since the Pka1 pathway and carbon source have not previously been described as modulators of SIN function. Still, the exact mechanism by which Pka1 intersects with the SIN has not been determined. The logic of the work and the data are clear. However, the paper suffers from a careless and somewhat disorganized presentation. If found suitable for JCS, I recommend condensing to a report format of 4 main display items and 4 supplementary items, particularly because no significant new information on etd1 function is presented and figures that essentially recapitulate previously published data could be placed in supplement. The main finding here is that cAMP levels and Pka1 modulate SIN function in a carbon source-dependent manner by an unknown mechanism and these data can be presented in 4 figures.

© 2023. Published by The Company of Biologists under the terms of the Creative Commons Attribution License (https://creativecommons.org/licenses/by/4.0/).

I have followed the referee's recommendation, reorganising the paper as described above.

Reviewer 2 Comments for the Author:
Major comments:

1. The manuscript deserves another round of thorough editing.
•As a first example, there is a word missing from the second sentence of the abstract and an extra word in line 42. ***This has been corrected***
•Word capitalization and use are inconsistent. Figure, figure; HIPPO, Hippo; Glucose, glucose; D, Δ; etc. ***I apologise for the poor copy-editing. I hope that I have managed to correct all the errors. The paper has been reread extensively by AK and OU and also shown to an external scientific editor.***
•The use of commas should be re-examined to improve readability. ***The point is well-made. I have reduced the number of semi-colon separated lists. I hope the new version is easier to read, despite the reduced length.***
•In the instance of multiple references in the text, they should be in the ***same format.*** This was due to a corruption of the endnote library, which has been remade.
•Considering references, there are some odd choices and omissions. As an example, Chan et al., 2017 is not an appropriate reference for the statement in the text that it is supposed to reference. Please re-examine referencing throughout. ***I have done so, expanding the referencing.***
•Labeling of figures should use nomenclature rules – lowercase italic for strains. wt or WT or wildtype for consistency. ***This is total word-blindness on my part, for which I apologise. I hope that everything now conforms to the required format and is uniform.***
•The introduction is too long. Some of the material on metabolism would be better suited to the results section where it would, in doses, set up the experimental design. There is also repetition as it stands. ***Agreed. I hope that everything is now mentioned only once.***
•Results are presented in figure legends (e.g. 2B, 4A, 4C) and should be instead only in the results section. ***I have complied with the request.***
•Line 738: compared
•Line 243. The sentence beginning in this line should be re-worded. It is clear what message is trying to be conveyed but the wording is not quite correct.
It is not known that the indicated mutant proteins are denatured at non-permissive temperature and this is what is predicted here. ***Agreed – I have eliminated this discussion from the text in the interests of space. As the referee rightly points out, there is no data arguing for or against total/partial denaturation of these mutant proteins.***
•The paragraph lines256-161 set up Figure 6, not Figure 5; it is in the wrong place in the manuscript. In general, the organization of what appears where needs to be scrutinized to remove redundancy and place information where it should be. ***My apologies for the poor copyediting. I hope that everything is now referred to appropriately.***
•The data showing images of Sid1 localization is missing. Line 365 refers the reader to Figure 5 but that does not show Sid1 localization. Sid1 localization is summarized in Figure 6, C and D and behaves as expected given the localization of the other components. ***The panel referencing error has been fixed.***
•Methods should be in methods. Some details are lacking. Spot assays as example – what dilutions? ***Dilutions have been provided in materials and methods.*** Tagging vectors were not used for transformation; PCR products were used. Etc. *The methods section has been checked for relevance/completeness. Methods for the generation of etd1-M9 have been added, for example.*

2. Figure 5 and 7B: I am unfamiliar with the definition of "compartmented". Does MN mean >2? Figure 5 and 7B would benefit from diagrams of what cells are being counted as what. ***Compartmented would indicated a cell with multiple compartments, most of which contain a nucleus, but where the septa have not cleaved. An example would be the pombe septin mutants, or the sep-family mutants. This is different to the multiseptated phenotype***

© 2023. Published by The Company of Biologists under the terms of the Creative Commons Attribution License (https://creativecommons.org/licenses/by/4.0/).

generated by (for example) cdc16-116, where there are one or two nuclei in a cell, and multiple septa. However, most "compartments" do not contain a nucleus.

Minor comments:

1. Scale bars are needed in Figure 1B and in 1C, it is not defined in the legend. Something is masking part of two images in Figure 1C. The quality of the most right-hand image in Figure 1C could be improved. Importantly, the conclusions would be bolstered by some quantitative data. ***See comment regarding figure 1/S1 above.***

2. A significant fraction of the world's population is red-green colorblind so to make the work more inclusive, I recommend using a magenta-green rather than red-green false color combination so that everyone can appreciate the images. ***We do not now have any micrographs that use colour.***

<u>Second decision letter</u>

MS ID#: JOCES/2023/261488

MS TITLE: A role for the cell's carbon source and Protein kinase A in regulating the S. pombe Septation Initiation Network.

AUTHORS: Ozge Uysal Ozdemir, Andrea Krapp, Bastien Mangeat, Marc Spaltenstein, and Viesturs Simanis
ARTICLE TYPE: Short Report

I am happy to tell you that your manuscript has been accepted for publication in Journal of Cell Science, pending minor changes requested by the reviewers and standard ethics checks. Referee reports are appended below.

<u>Reviewer 1</u>

Advance summary and potential significance to field

The fission yeast SIN represents a remarkably well characterised signal transduction cascade whose spatial control sits at the heart of the regulation of cytokinesis in fission yeast and, by virtue of the conservation of function in budding yeast and neurospora is assumed to do so throughout fungi. As such, extending insight into the SIN regulatory network will be of interest to the broad readership of Journal of Cell Science. Etd1 is one of the least understood components of the SIN and so this manuscript is addressing a key missing piece of the SIN jigsaw. Through a pathway of genetic analyses Ozdemir et al show how Etd1 function is tied in to carbon metabolism and reveals a major contribution of cAMP signalling to SIN control. The conclusions are consistent with the data and I am supportive of publication.

Comments for the author

The revised manuscript is much improved, more accessible and the greater focus communicates the principal message of the story much more clearly and succinctly than the previous version. I am therefore very supportive of publication in this revised form.

minor points

- Line 71 - Grallert et al. 2004 10.1101/gad.296204 should be inserted as the reference that demonstrates Cdc7 recruitment to the new SPB
- Microsoft word has worked its magic on the genetic nomenclature on supplementary figures 1 and 2 in converting the first letter of the first gene in a genotype to upper case.

© 2023. Published by The Company of Biologists under the terms of the Creative Commons Attribution License (https://creativecommons.org/licenses/by/4.0/).

- Line 183 –Figure S3F should be changed to Figure S3G as S3F is all about the SPB and S3G about the CAR

Reviewer 2

Advance summary and potential significance to field

The authors have satisfied my major concerns by clarifying and re-organizing the text. The findings will be of interest to those studying yeast metabolism and cytokinesis.

Comments for the author

There remain a few text issues:

Line 176 has an extra word (in) or is missing an extra word.

Line 183: I think the text should refer to Figure S3G, not S3F.

In terms of Figure S3, the authors might consider re-organizing so that the top of the figure is discussed first in the text.

© 2023. Published by The Company of Biologists under the terms of the Creative Commons Attribution License (https://creativecommons.org/licenses/by/4.0/).
